# Structure Design Optimization of a Differential Capacitive MEMS Accelerometer Based on a Multi-Objective Elitist Genetic Algorithm

**DOI:** 10.3390/mi17010129

**Published:** 2026-01-19

**Authors:** Dongda Yang, Yao Chu, Ruitao Liu, Xiwen Zhang, Saifei Yuan, Fan Zhang, Shengjie Xuan, Yunzhang Chi, Jiahui Liu, Zetong Lei, Rui You

**Affiliations:** 1 College of Instrument Science and Opto-Electronics Engineering, Beijing Information Science and Technology University, Beijing 100192, China; yangdongda@bistu.edu.cn (D.Y.); zhangfan@bistu.edu.cn (F.Z.); leizetong1120@163.com (Z.L.); 2 State Key Laboratory of Precision Measurement Technology and Instruments, Tsinghua University, Beijing 100095, China; liuruitao_2008@sina.com (R.L.); zhangxiwen@semi.ac.cn (X.Z.); yuasaifei@163.com (S.Y.); shengjiex@outlook.com (S.X.); chiyunzhang4@gmail.com (Y.C.); liujiahui.t@outlook.com (J.L.)

**Keywords:** multi-objective optimization, global structure design, elitist genetic algorithm, MEMS accelerometer

## Abstract

This article describes a global structure optimization methodology for microelectromechanical system devices based on a multi-objective elitist genetic algorithm. By integrating a parameterized model with a multi-objective evolutionary framework, the approach can efficiently explore design space and concurrently optimize multiple metrics. A differential capacitive MEMS accelerometer is presented to demonstrate the method. Four key objectives, including resonant frequency, static capacitance, dynamic capacitance, and feedback force, are simultaneously optimized to enhance sensitivity, bandwidth, and closed-loop driving capability. After 25 generations, the algorithm converged to a uniformly distributed Pareto front. The experimental results indicate that, compared with the initial design, the sensitivity-oriented design achieves a 56.1% reduction in static capacitance and an 85.5% improvement in sensitivity. The global multi-objective optimization achieves a normalized hypervolume of 35.8%, notably higher than the local structure optimization, demonstrating its superior design space coverage and trade-off capability. Compared to single-objective optimization, the multi-objective approach offers a superior strategy by avoiding the limitation of overemphasizing resonant frequency at the expense of other metrics, thereby enabling a comprehensive exploration of the design space.

## 1. Introduction

Microelectromechanical system (MEMS) devices are widely used in various areas such as aerospace, industrial monitoring, and consumer electronics, due to their small size, light weight, and low cost [1]. The vast majority of MEMS devices, including accelerometers [2,3], gyroscopes [4], and actuators [5,6], consist of multiple substructures, such as proof masses, beams, and comb fingers. MEMS devices are typically designed in a sequential manner, where each substructure is manually designed and subsequently assembled into a complete device [7]. For instance, in a conventional MEMS accelerometer design, the beam structure is first manually defined, followed by the design of the comb fingers and the proof mass to meet specific requirements. Such a design method prevents a MEMS device from reaching its full potential. As the components of a MEMS device are intrinsically coupled, the device cannot achieve its best performance by simply combining individually optimized components. Such a design process is time-consuming, involves multiple rounds of trial and error, and is technically cumbersome, requiring considerable specialized expertise [8]. Additionally, the conventional manual design method is not generic and can only handle specific or well-understood MEMS devices [9].

To overcome these limitations, researchers have employed simplified models to describe the overall structure of MEMS devices and conduct optimization. Chen et al. integrated the second-order model of a MEMS gyroscope into the system-level model of a sigma-delta modulator (SDM) and developed a double closed-loop control system for a MEMS gyroscope [10]. However, these models often oversimplify the mechanical behavior and fail to capture aspects such as higher resonant modes [11] of MEMS accelerometers and the quadrature coupling [12] in MEMS gyroscopes. Meanwhile, designers often adopt a single-objective optimization strategy during optimization process [6], whereas MEMS device design usually requires trade-offs among multiple performance metrics, such as sensitivity, bandwidth, and measurement range. Wang et al. used a single-objective optimization algorithm to perform electro-mechanical co-optimization on the system-level model of the MEMS accelerometer [13]. In single-objective optimization, it is necessary to construct an objective function that integrates multiple performance metrics into a figure of merit (FOM), allowing the algorithm to search for the optimum automatically. The FOM is usually formulated in a ratio or product form. For performance metrics with different value ranges, the result of single-objective optimization strongly depends on the design of the FOM, which may easily lead to a local optimum dominated by individual performance metrics.

Therefore, although some MEMS devices have demonstrated superior performance in structure design, there remains no design methodology capable of performing global optimization over the whole structural parameter space while simultaneously and independently optimizing multiple key performance metrics, which fundamentally limits the achievable performance of MEMS devices. This is especially important for MEMS devices with complex mechanical structures and nonlinear characteristics, as the coupling effects between substructures are not sufficiently accounted for by conventional design methods. This article describes a multi-objective global automatic optimization methodology for MEMS devices. The proposed methodology can globally optimize the substructure design of a MEMS device to achieve improvements in multiple performance metrics, including bandwidth, sensitivity, and measurement range, without human intervention, thereby providing a new working pipeline for the design of MEMS accelerometers. Also, this methodology can be extended to various MEMS devices and does not require significant computation time. The proposed design methodology is demonstrated by a case study on a differential capacitive MEMS accelerometer.

## 2. Optimization Methodology Based on a Genetic Algorithm

**Figure 1:**
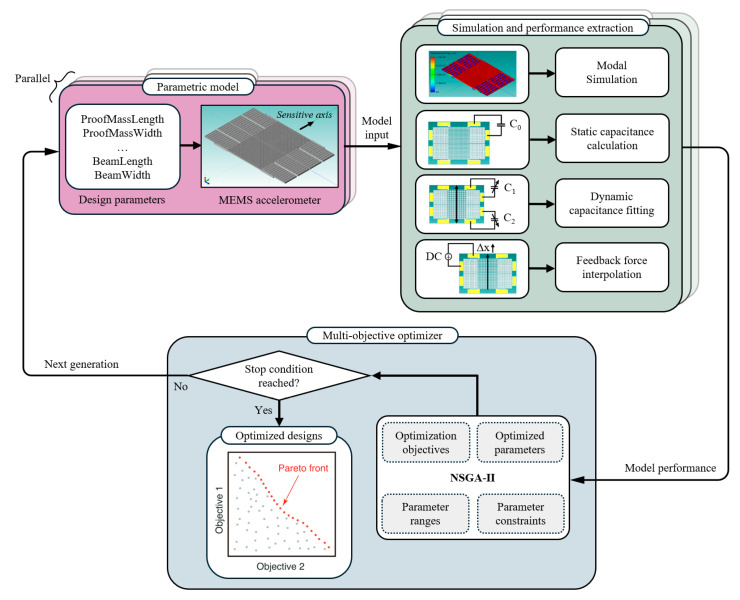
Global structure design optimization method based on a multi-objective elitist genetic algorithm.

As shown in Figure 1, the proposed design methodology consists of three components: (i) a parametrized model of the structure developed in Coventor MEMS+ 7.1, (ii) an automated simulation and data-processing framework in MATLAB R2021b for performance extraction, and (iii) a multi-objective elitist genetic algorithm (NSGA-II) implemented in MATLAB for optimization. First, a fully parameterized structure model of the device is constructed in MEMS+ to serve as the foundation for optimization. This follows a modular approach by simply selecting and connecting pre-validated components in an intuitive 3D graphical interface. Each component is supported by an underlying behavioral model that enables parameterization and can be accessed and modified through the MATLAB scripting interface. Thus, with the optimization toolbox in MATLAB, an automated optimization process based on the multi-objective elitist genetic algorithm can be achieved by updating the MEMS+ model parameters through a script. It should be noted that the parameter values during optimization need to be as widely distributed as possible across the entire design space to ensure convergence toward a global rather than a local optimum. As a global parameterized model has a vast number of degrees of freedom, a simple nested sweep of all parameters would result in a prohibitive computational cost. In contrast, the multi-objective elitist genetic algorithm can search for a global optimum without parameter scanning. This avoids convergence to local optima and provides advantages over other methods, such as a gradient descent algorithm [14].

### 2.1. Parameterized Modeling of MEMS Accelerometer

**Figure 2:**
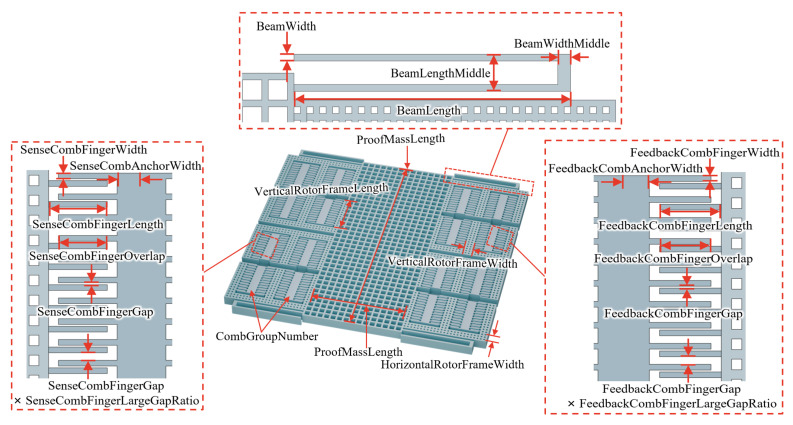
Parametric structure model of the capacitive MEMS accelerometer.

The MEMS accelerometer used in this study is shown in Figure 1 and Figure 2. It mainly consists of a proof mass, suspension beams, sensing combs (for differential capacitive readout), and feedback combs (for applying electrostatic forces). When the device is subjected to acceleration along its sensitive axis, the proof mass is displaced in the same direction, causing relative motion between the fixed and movable fingers of the sensing combs. This motion alters the finger gaps and consequently results in differential changes in capacitance [15]. Open-loop acceleration measurement can be realized by detecting the differential variation in capacitance. When a DC voltage is applied to the feedback combs, an electrostatic force opposite to the inertial force is generated to counteract the displacement caused by acceleration, which is the closed-loop working mode of the accelerometer [16]. At this point, the applied DC voltage serves as a measure of the input acceleration. However, the closed-loop performance strongly depends on the structure design of the device. In this work, a global optimization of the substructure design of a MEMS accelerometer is conducted, combining it with a multi-objective optimization strategy, to achieve balanced trade-offs among multiple performance metrics and to obtain superior design solutions that are adaptable to different application requirements.

The structure model must be fully parameterized to enable global optimization. This means that all relevant geometric parameters must be defined as variables rather than fixed values. Moreover, appropriate value ranges and constraints must be specified for all structural parameters. For the MEMS accelerometer used in this study, a total of 22 parameters is defined to characterize its structure topology. These parameters define the dimensional boundaries of the proof mass, suspension beams, sensing combs and feedback combs, as well as the number of comb groups.

It should be noted that in capacitive accelerometers, some key structures, such as suspension beams and combs, are critical to overall performance. These structures influence not only the stiffness of the system but also the capacitive output, directly affecting the device sensitivity [16]. To accurately capture the coupling effects of complex structures, device models can be constructed with the aid of commercial simulation tools such as COMSOL 6.3 and Coventor MEMS+ 7.1. In this article, Coventor MEMS+ 7.1 was chosen due to its compatibility with MATLAB R2021b and the capability for co-simulation with Simulink. Also, the MEMS+ model considers mechanical dynamic nonlinear and squeeze-film damping to accurately characterize the dynamic behavior of the device.

The parametric modeling process of the differential capacitive MEMS accelerometer is shown in Figure 2, and its structure features are fully defined by the parameters listed in Table 1. For fabrication convenience, all parameter values were constrained to integers during the optimization process. This set of parameters fully characterizes the structure of the accelerometer, laying the foundation for optimization. The mapping between structural parameters and geometric features of the model is shown in Figure 2, illustrating how the structural parameters define the device geometry.

The 22 parameters listed in Table 1 are defined with lower and upper bounds (LBs and UBs, respectively) determined either (i) by fabrication rules [17] or (ii) by a qualified guess by the designer of the optimum value. For example, the minimum gap between comb fingers is constrained by the fabrication limits of deep reactive ion etching (DRIE), while other parameters are determined based on prior design experience. A reasonable definition of parameter ranges not only ensures the manufacturability of the device but also improves the convergence efficiency of the optimization algorithm. Specifically, *W_VF_*, *W_SA_*, and *W_FA_* in Table 1 are set as constants, meaning that they are not involved in the automatic optimization, which helps reduce the computational overhead. In addition, it is necessary to introduce constraints on the design parameters during the parameterized modeling process. The constraints are established based on three considerations: (i) process constraints, to ensure the device is manufacturable; (ii) topological relationships, to guarantee model construction without geometric conflicts; and (iii) design experience, to reduce the infeasible solutions during the optimization process. As listed in Table 2, a total of 13 constraints are defined in this article. Among these, constraints (1)–(5) come from design experience; constraints (8)–(11) come from geometric relationships; and constraints (6), (7), (12) and (13) come from process limitations.

### 2.2. Optimization Objectives

In capacitive MEMS accelerometers, capacitance is a key metric that determines the sensitivity, signal-to-noise ratio, and overall device performance. The capacitance metrics include the static capacitance C_0_ and the dynamic capacitance ΔC. The static capacitance C_0_ refers to the intrinsic capacitance between a pair of detection electrodes. An excessively large C_0_ reduces the dynamic range and increases the thermal noise level in the readout circuit. The dynamic capacitance ΔC represents the capacitance variation induced by external acceleration. A larger ΔC indicates a stronger electromechanical transduction response of the device to the applied acceleration. In addition, the ratio of dynamic to static capacitance (ΔC/C_0_) is commonly used to characterize the sensitivity of a capacitive MEMS accelerometer [18,19]. Ideally, the device should have both high sensitivity and wide bandwidth, with the latter primarily determined by the first-order modal resonant frequency *f_y_*. Therefore, in this article, the first-order modal resonant frequency *f_y_*, static capacitance C_0_, and dynamic capacitance ΔC are defined as the optimization objectives. In addition, for this accelerometer, closed-loop operation offers superior performance compared to open-loop. As the electrostatic force applied by the feedback combs can counteract the proof mass displacement induced by external acceleration, maintaining it at the equilibrium position [20]. This can reduce output nonlinearity, extend the dynamic range, and enhance the overall stability of the device. The closed-loop performance depends on a sufficient feedback force; therefore, the feedback force F_b_ is also defined as an optimization objective.

In summary, four optimization objectives are defined in this article: (i) the first-order modal resonant frequency, expressed as |*f_y_* − 1300|, which aims for a target value close to 1300 Hz and (ii) the static capacitance C_0_, to be minimized and (iii) the dynamic capacitance ΔC, to be maximized and (iv) the feedback force F_b_, calculated under a 5 V DC voltage bias, also to be maximized. A global optimization is conducted in this article based on a multi-objective elitist genetic algorithm, which enables the four objectives to be optimized independently and simultaneously within the same process without constructing a function of four objectives, thereby generating a Pareto solution set that balances the multiple performance metrics. It should be noted that the reference values of 1300 Hz used for the objectives of resonant frequency are selected based on the following considerations [21]. The resonant frequency of the MEMS accelerometer is usually 1–2 kHz. Based on the original design of 1146 Hz, this study targets an increased resonant frequency of 1300 Hz to improve the bandwidth. For the feedback force simulation, a 5 V bias voltage was applied. This value was selected because MEMS sensors typically operate at low power and within limited voltage ranges. Adopting a 5 V DC bias aligns with the standard operating voltage commonly used in both sensor interfaces and on-chip integrated circuits, ensuring consistency with practical implementations.

### 2.3. Optimization Algorithm

In this work, a multi-objective elitist genetic algorithm is employed, which is based on the principles of natural selection and genetics and is capable of simultaneously optimizing multiple conflicting objectives [22,23]. Such evolutionary algorithms excel in handling high-dimensional optimization problems and can efficiently yield optimal solution sets. During the optimization process, the algorithm first generates an initial population, where each individual corresponds to a set of model parameters that are subsequently simulated. The performance metrics are then extracted from the simulation results and used as optimization objectives.

The algorithm constantly updates the population through crossover, mutation, and elite retention. By introducing an elitist selection strategy, the best individuals of each generation are preserved, so that the algorithm converges to the global Pareto front. After several generations of iteration, the population gradually converges to a set of non-dominated solutions, known as the Pareto front. The designer can then select an optimal set of design parameters from these solutions according to specific application requirements. Previous studies have primarily focused on individual structure or used single-objective optimization, without performing multi-objective global optimization to explore the whole design space. Therefore, this study integrates a parameterized model with a multi-objective optimization strategy to globally search the design parameters, achieving balanced trade-offs among multiple performance metrics and improving the overall device performance.

## 3. Optimization Process and Results

### 3.1. Optimization Process

In the first step of the optimization process, the multi-objective elitist genetic algorithm generates 800 individuals as the initial population, which are uniformly distributed within the parameter ranges in Table 1 while satisfying the constraints specified in Table 2. For each individual, a fully parameterized structure model is constructed, and a simulation analysis is performed. The simulation includes four parts: (i) performing a modal analysis to obtain the resonant frequency; (ii) calculating the static capacitance of the sensing combs; (iii) sweeping the acceleration from −1 g to +1 g to obtain the acceleration-versus-differential capacitance curve; and (iv) conducting an acceleration sweep under a 5 V bias voltage to determine the equivalent acceleration when the accelerometer is at its balanced position. Subsequently, the simulation results are imported into MATLAB to extract the optimization objectives. The first-order modal resonant frequency *f_y_* and static capacitance C_0_ can be directly obtained from the simulation results. The dynamic capacitance ΔC is obtained by fitting the interval with a linearity of not less than 99% in the acceleration-differential capacitance curve and calculating its slope. The feedback force is obtained by interpolating the acceleration-displacement curve to determine the acceleration corresponding to zero proof mass displacement. It is worth noting that both the simulations and objective extraction in this study employ a parallel strategy, in which multiple individuals within the same generation are simulated and evaluated simultaneously. This significantly improves the optimization efficiency, reducing the computation time by more than one order of magnitude compared with the conventional serial strategy.

After the evaluation of the initial population, the algorithm proceeds to the iterative stage. For each generation, the algorithm first performs non-dominated sorting to classify the individuals into several ranks and then calculates the crowding distance among individuals within the same rank. Next, individuals with higher ranks and larger crowding distances are preferentially selected as parents, and offspring individuals are generated through crossover and mutation operations. The algorithm evaluates the performance of all offspring individuals through simulation analyses. Subsequently, the algorithm merges the offspring and parent populations, after which non-dominated sorting is reapplied, and the crowding distance is recalculated. Finally, the algorithm applies the elitist selection strategy to the merged population, selecting the next generation based on the updated ranks and crowding distances. This process ensures the preservation of superior individuals while maintaining population diversity.

**Figure 3:**
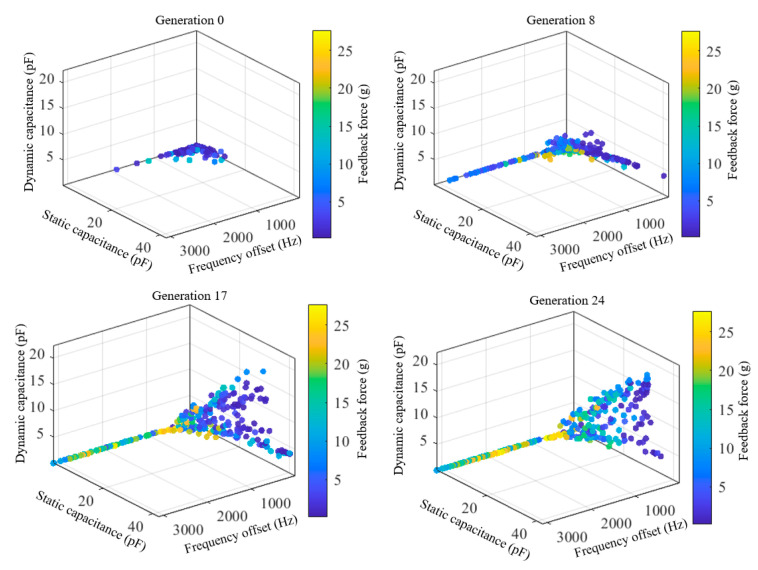
Evolution of the Pareto front in multi-objective global optimization.

**Figure 4:**
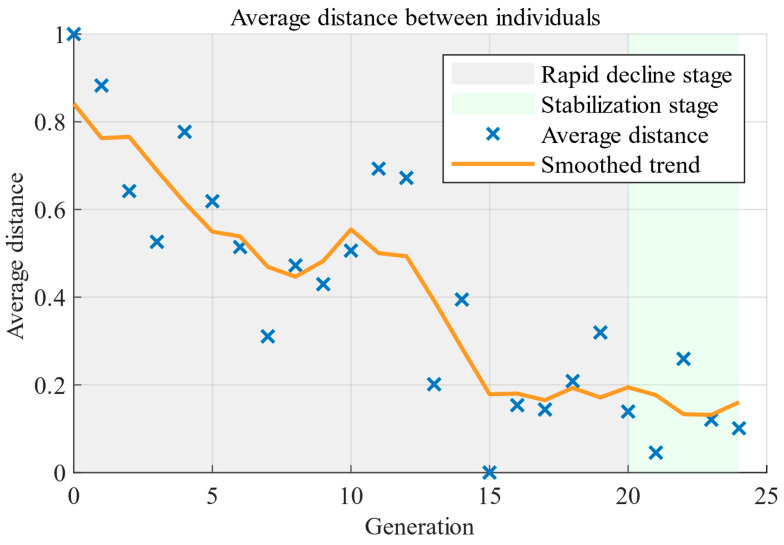
The trend of the average distance between individuals at each generation.

Figure 3 illustrates the evolution of the Pareto front during the iterative process. The initial Pareto front is mainly distributed near the boundaries of the objective space, and an effective front has not yet formed. As the iteration progresses, the solution set gradually expands and covers a larger region of the objective space, indicating that the algorithm effectively explores the design space. As observed in the generation 8 and 17 shown in Figure 3, the distribution of solutions becomes more uniform, and the Pareto front gradually takes shape, indicating that the algorithm transitions from the exploration phase to the convergence phase, where the Pareto front tends to stabilize. By the generation 24, a complete and uniformly distributed Pareto front is obtained, clearly illustrating the trade-offs among different performance metrics. Figure 4 illustrates the trend of the average distance between individuals at each generation. It reflects the dispersion of the population in the design space and can be used to characterize the convergence state of the algorithm. As shown in Figure 4, the average distance decreases rapidly in the early iterations, indicating that the algorithm performs an extensive global search during this stage. Subsequently, the rate of decrease in the average distance gradually slows down and stabilizes, indicating that the algorithm has entered the convergence phase, with the population distribution becoming stable. This trend indicates that the optimization process has good convergence and stability.

### 3.2. Optimization Results

**Figure 5:**
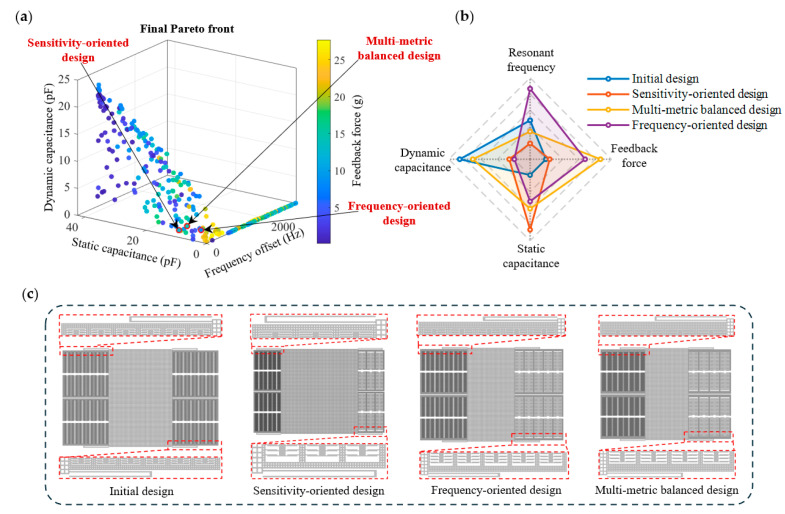
Global optimization design results of MEMS accelerometer based on NSGA-II. (**a**) The final Pareto front and the position of the selected representative solution in it; (**b**) Performance comparison radar chart of the initial design and three representative designs. The outermost circle denotes better performance; (**c**) The structure of the initial design and three representative solutions.

For the designs presented in this article, the optimization process ran continuously for 25 generations, with each generation having a population of 800 individuals. On a high-performance computing server equipped with an Intel processor, approximately 20,000 parameterized models were evaluated, with a total computation time of about 11.5 h. Figure 5 illustrates the final Pareto front for the four-objective optimization. The results clearly demonstrate the inherent conflicts among the optimization objectives. Solutions with smaller static capacitance are generally accompanied by a reduction in dynamic capacitance, whereas those exhibiting larger dynamic capacitance can improve sensitivity but inevitably lead to an increase in static capacitance. In practice, different application fields place distinct emphasis on specific performance aspects. For instance, in geophysical surveying and gravity monitoring, exceptional sensitivity is often prioritized to achieve nano-g-level resolution, enabling high-precision observation of crustal deformation and seismic activities [24,25]. In industrial vibration monitoring, a broad operational bandwidth is emphasized to enhance impact resistance and ensure reliable performance under harsh dynamic conditions [26,27]. In biomedical applications, such as intelligent implantable hearing aids, achieving a balance among sensitivity, power consumption, and device size is essential to ensure both robustness and practicality [28,29]. Therefore, extreme optimization of a single metric is often undesirable; instead, it is more practical to select representative solutions from the Pareto front according to specific application requirements.

To further demonstrate the flexibility of this optimization approach, three representative designs with distinct performance characteristics were selected from the Pareto front. The first is a sensitivity-oriented design with large dynamic capacitance and low static capacitance, leading to a stronger open-loop output signal. The second is a high resonant frequency design that provides a wider bandwidth while maintaining reasonable capacitance levels. The third is a multi-metric balanced design that exhibits moderate performance in four objectives, achieving a trade-off among sensitivity, bandwidth, and feedback force. The parameter values of the three representative designs are listed in Table 3. It is worth noting that no fixed constraints were imposed on capacitance metrics during the optimization process; instead, only optimization directions were specified to preserve the diversity of solutions. To match the interface circuit used in this study, a selection window was introduced during the design screening stage: (i) static capacitance C_0_ ≤ 10 pF, (ii) dynamic capacitance ΔC ≥ 1 pF. This window was not used as an optimization constraint but rather as a selection criterion based on the experimental conditions of this study. The positions and structural models of the three representative solutions in the Pareto front are shown in Figure 5a. Compared with the initial design, the geometric adjustments of the three representative solutions reflect distinct optimization directions and their underlying physical mechanisms.

Table 4 lists the performance metrics of the initial design and the three representative solutions and compares the differences in each performance metric. The radar chart in Figure 5b visually illustrates the differences and trade-offs among the performance metrics. The initial design is based on a typical capacitive MEMS accelerometer and was manually defined by an expert according to established design rules. For the sensitivity-oriented design, a reduction in the total area of the sensing combs combined with an optimized fingertip gap effectively suppressed the static capacitance C_0_ to 4.93 pF while keeping the dynamic capacitance above ΔC 1 pF. Consequently, the ΔC/C_0_ ratio was improved by 82.1%, resulting in a substantial enhancement in sensitivity, whereas the first-order modal resonant frequency *f_y_* decreased by only 16.1%. For the frequency-oriented design, the proof mass dimensions were reduced and the stiffness of the suspension beams was reinforced, thereby decreasing the effective mass while increasing the structural rigidity. As a result, the characteristic frequency increased by 22.1% to 1398.8 Hz, accompanied by a 66.4% increase in feedback force. Despite a slight decrease in sensitivity, the design exhibits a higher bandwidth and stronger closed-loop driving capability. For the multi-metric balanced design, a coordinated optimization of the overall structure achieved a trade-off among the four performance metrics: the first-order modal resonant frequency remained above 1 kHz, the feedback force increased significantly by 92.3%, and the ΔC/C_0_ ratio improved by 51.7% compared with the initial design. Overall, the design exhibits enhanced versatility and robustness.

## 4. Experiments and Results

### 4.1. Device Fabrication

**Figure 6:**
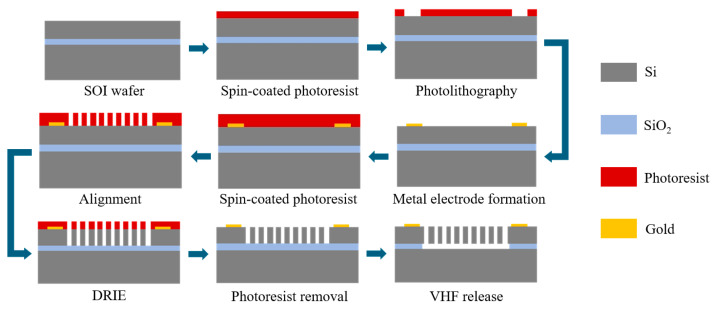
Fabrication process flow of the MEMS accelerometers.

To verify the effectiveness of the proposed optimization method, the initial design and the sensitivity-oriented design are selected as representative devices for fabrication. The selection of these two designs for experimental validation is primarily determined by the limitations of the current experimental platform. Therefore, the experimental section focuses on static capacitance and open-loop sensitivity. The devices fabrication process is described as follows. The silicon-on-insulator (SOI) technology used in the fabrication of the MEMS accelerometers is illustrated in Figure 6. The SOI wafer utilized has a 60-μm-thick device layer with an orientation of <100>, P-type silicon and a resistivity of 0.001 ohm.cm. The buried oxide (BOX) layer is 2-μm-thick, and the handle layer is 400-μm-thick. First, a 2-μm-thick photoresist is spin-coated on the wafer surface, and the electrode patterns are transferred onto the wafer by photolithography. Subsequently, 40 nm of chromium and 300 nm of gold are deposited by electron-beam evaporation. The metal electrode is finally formed by a lift-off process. Next, a 7-μm-thick photoresist is spin-coated, and the device patterns are transferred onto the wafer by alignment photolithography. The device layer is then etched by deep reactive ion etching (DRIE) until it is completely etched through. After, the photoresist is removed. Finally, the buried oxide (BOX) layer is etched using vapor phase hydrofluoric acid (VHF) to release the movable structures of the accelerometer.

**Figure 7:**
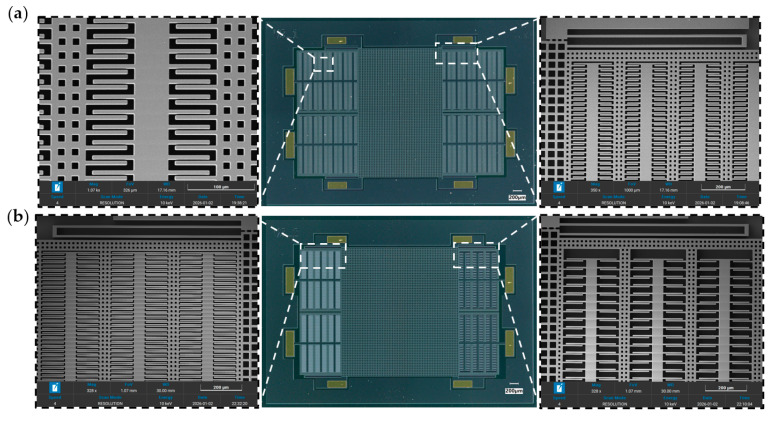
Fabrication results of the MEMS accelerometers. (**a**) Optical microscope and SEM images of the initial design; (**b**) Optical microscope and SEM images of the sensitivity-oriented design.

The fabrication results of the two designs are illustrated in Figure 7. The critical regions of the devices are characterized by scanning electron microscope (SEM). The proof mass, suspension beams, and comb fingers are clearly observed. The SEM results indicate that the key geometrical features of both devices are consistent with the designed layouts, confirming their manufacturability.

### 4.2. Experimental Step and Results

**Figure 8:**
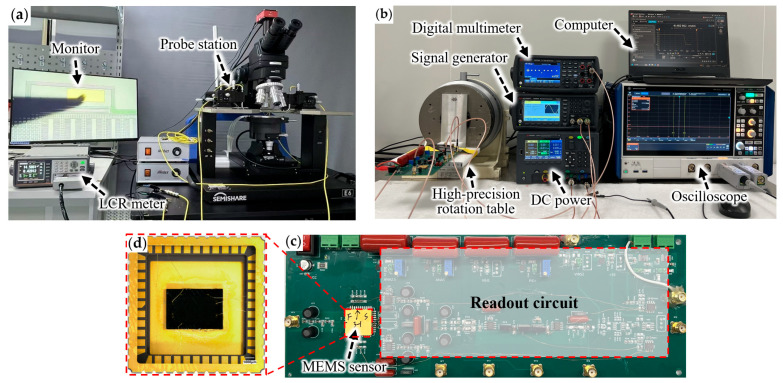
Measurement setup. (**a**) Measurement system for the measurement of static capacitance; (**b**) Measurement system for the measurement of static acceleration input; (**c**) Accelerometer readout circuit; (**d**) Accelerometer package diagram.

The static capacitance measurement setup for the MEMS accelerometer is shown in Figure 8a. The MEMS device was placed on a probe station, where one end of the probe contacted the device electrodes and the other end was connected to an LCR meter. The static capacitance of the accelerometer was measured under zero external input conditions. Table 5 lists the measured static capacitance results of the two designs. The experimental results show that, compared with the initial design, the static capacitance of the sensitivity-oriented design is significantly reduced from 18.78 pF to 8.24 pF, with a reduction of 56.1%. The experimental results exhibit a consistent variation trend with the simulation analysis, thereby validating the reliability of the simulation model.

**Figure 9:**
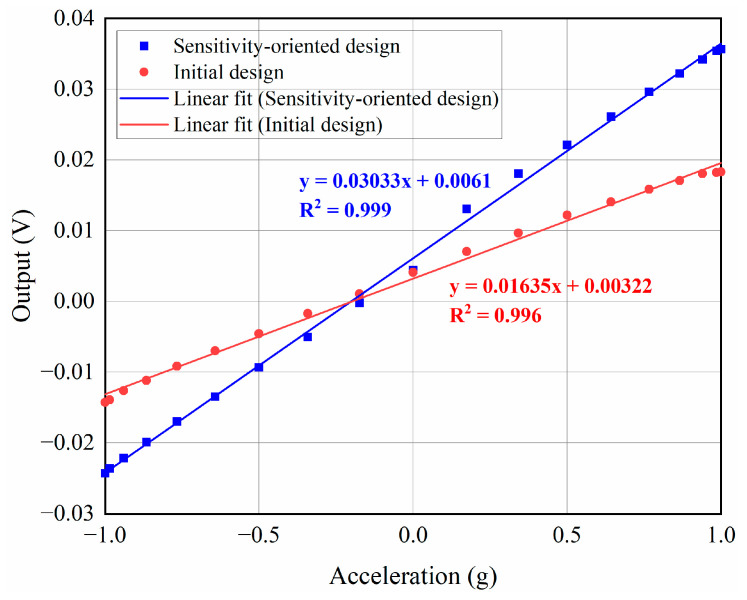
Output voltage responses for a range of ±1 g. Linear fitting is performed using the least-squares method.

To compare the sensitivity of the two designs, the experimental system shown in Figure 8b is used to measure the output signals of the two MEMS accelerometers for a range of ±1 g. To ensure the comparability of the experimental results, both designs are measured under identical conditions using the capacitive readout circuit shown in Figure 8c. The measurement results of the two designs are shown in Figure 9. According to the linear fit equations shown in Figure 9, the sensitivity (scale factor) of the initial design is 16.35 mV/g with the R^2^ is 0.996. While the sensitivity-oriented design has a sensitivity of 30.33 mV/g with the R^2^ is 0.999. Compared with the initial design, the sensitivity of the sensitivity-oriented design is increased by 85.5%. The experimental results demonstrate that the proposed optimization method can significantly improve the device sensitivity while maintaining good linearity.

## 5. Discussion

### 5.1. Comparison of Different Optimization Methodologies

#### 5.1.1. Multi-Objective Local Optimization

To demonstrate the superiority of the proposed global optimization methodology, a multi-objective optimization of local structures was performed under identical conditions, including the optimization parameters, parameter ranges, constraint definitions, and algorithm configurations. To ensure a fair comparison, the local optimization employed the same population size of 800 individuals per generation and the same total number of 25 generations as the global optimization. The local optimization was categorized into three types: (i) optimization of the beams only; (ii) optimization of the proof mass only; and (iii) optimization of the combs only. When one substructure was optimized, the others were kept fixed as in the initial design.

**Figure 10:**
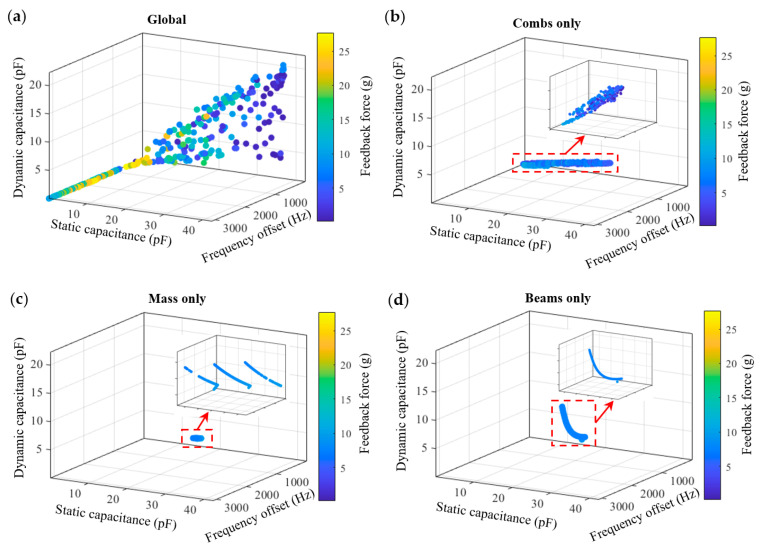
Pareto front for global and local structure optimization. (**a**) Global structure optimization Pareto front; (**b**) Combs only optimization Pareto front; (**c**) Mass only optimization Pareto front; (**d**) Beams only optimization Pareto front.

Figure 10 compares the Pareto fronts obtained from the global and local optimizations. It can be observed that the global optimization exhibits the widest distribution of solutions and the greatest coverage in the design space, forming a continuous and well-extended Pareto front. In contrast, all three local optimization results exhibit pronounced limitations. The solutions from the optimization of the proof mass only and the optimization of the beams only are both concentrated in regions of low sensitivity and small dynamic capacitance. Since adjusting the two structures mentioned above cannot modify the design parameters of combs, the tunable range of the dynamic capacitance is extremely limited, making it difficult to enhance the sensitivity effectively. Moreover, since the electrostatic feedback force originates from the comb fingers, its magnitude cannot be significantly enhanced without optimizing the comb finger design. Although these two local optimizations exhibit an improvement in the resonant frequency, their overall Pareto fronts remain narrow and fail to achieve a well-balanced trade-off among multiple objectives. Similarly, the solutions from the optimization of the combs are mainly distributed along the static capacitance axis, forming an approximately planar Pareto front. This suggests that although tuning the comb parameters alone can improve the capacitive performance, the frequency enhancement is limited due to the fixed proof mass and beam structures.

**Figure 11:**
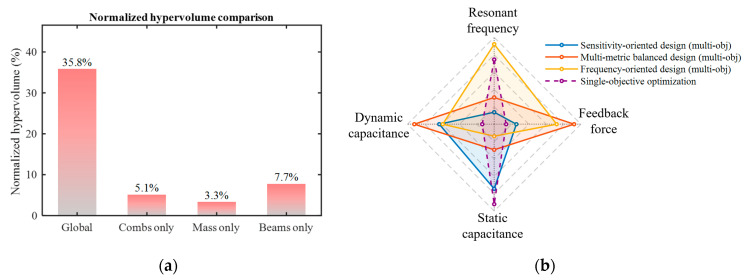
Comparison of metrics for various optimization strategies. (**a**) Normalized hypervolume with different optimization strategies; (**b**) Performance comparison radar chart of representative designs from different optimization strategies. The outermost circle denotes better performance.

Figure 11a shows the comparison of normalized hypervolume (HV) among different optimization strategies. The hypervolume indicator quantifies the volume of the objective space dominated by the Pareto front, reflecting both the convergence and diversity of the solution set. At the same reference point, a larger HV value indicates that the optimization results cover a broader region of the objective space, thereby providing a richer set of trade-off solutions. As shown in Figure 11a, the global optimization shows the highest HV value of 35.8%, significantly higher than that of the local optimization strategies. This indicates that local optimization, constrained by limited structural parameters, fails to capture the coupling effects among components, resulting in a solution set that cannot form a truly non-dominated expansion in the objective space. In contrast, the global optimization strategy enables the collaborative tuning of multiple structural parameters, significantly expanding the design space. This allows the resulting solution set to exhibit enhanced diversity and trade-off characteristics, thereby providing more effective solutions for the design of coupled structures.

#### 5.1.2. Single-Objective Global Optimization

To further demonstrate the superiority of the proposed global optimization methodology, a single-objective global structure optimization was performed under identical conditions, including the optimization parameters, parameter ranges, constraint definitions, and algorithm configurations. To ensure a fair comparison, the single-objective optimization employed the same population size of 800 individuals per generation and the same total number of 25 generations as the global optimization. Since single-objective optimization involves only one objective, all goals were integrated into a unified composite function, defined as follows:

(1) $$FOM=\frac{\left|f_{y}-1300\right|}{F_{b}}\cdot \frac{C_{0}}{\Delta C}$$

where FOM is the value actually optimized by the algorithm. During the optimization, the algorithm searches for the minimum FOM, thereby indirectly optimizing each objective in the function. As the single-objective optimization only yields a unique optimal solution with the minimum FOM. For intuitive comparison, the top ten optimization results ranked by ascending FOM are listed in Table 6, with the first solution representing the optimal result of the single-objective optimization. A radar chart comparing the optimal solutions of the single-objective and multi-objective optimizations is shown in Figure 11b.

The results indicate that although the single-objective optimization achieves convergence in FOM, merging all objectives into one function prevents the algorithm from independently controlling the optimization direction of each objective, leading to a bias toward the objectives that are easier to optimize. For example, as shown in Table 6, the optimized designs exhibit a noticeably reduced dynamic capacitance ΔC, while the frequency offset |*f_y_* − 1300| is excessively minimized. This suggests that single-objective optimization tends to sacrifice part of the performance to minimize the FOM, exhibiting poor balance among multiple objectives and a high risk of falling into local optima in complex design spaces. In contrast, multi-objective optimization treats each metric as an independent objective and optimizes them simultaneously, thereby explicitly capturing the trade-off relationships among different metrics. This approach prevents the optimization results from being dominated by a single metric, enables a comprehensive exploration of the design space. And yields a set of non-dominated solutions that are well-balanced across multiple performance dimensions.

### 5.2. Results Analysis

This work proposes a global multi-objective optimization methodology for MEMS devices and demonstrates it by a capacitive MEMS accelerometer. The experimental results show that, compared with the initial design, the optimized design (sensitivity-oriented design) achieves a 56.1% reduction in static capacitance and an 85.5% improvement in sensitivity. These results demonstrate the multi-objective optimization capability of the proposed approach with respect to sensitivity-related performance. Owing to the limitations of the current experimental conditions, the remaining performance metrics are evaluated at the simulation level. With further improvements in the experimental conditions, future studies will consider conducting measurements of bandwidth and dynamic range, as well as performing a more detailed analysis of the electromechanical coupling characteristics between the device and the interface circuit.

It should be noted that certain discrepancies exist between the experimental results and the simulation results. This may be caused by the following reasons: device structure asymmetry and critical dimension loss due to process errors, the parasitic capacitance effects. For example, parasitic capacitance between the metal electrodes and the substrate silicon, as well as that introduced by the measurement cables. Nevertheless, the experimental results still support the effectiveness of the proposed optimization methodology.

## 6. Conclusions

This paper presents a global optimization methodology for MEMS devices, which integrates a multi-objective elitist genetic algorithm for comprehensive performance exploration. By integrating parameterized modeling with a genetic algorithm, efficient multi-objective optimization of complex MEMS structures is achieved. A case for a differential-capacitive MEMS accelerometer with a globally parameterized model was used to demonstrate the validity of the proposed methodology. The results show that the method can effectively explore the high-dimensional design space and yields a uniformly distributed, continuous set of Pareto fronts. Compared with local and single-objective optimization, the global multi-objective approach exhibits superior capability in design space exploration and performance trade-off. The experimental results indicate that the optimized sensitivity-oriented design achieves a 56.1% reduction in static capacitance and an 85.5% improvement in sensitivity compared with the initial design. This validates the effectiveness of the proposed method in multi-objective coupled design, offering a new approach for the automated structure optimization of complex MEMS devices.

In addition to the multi-objective elitist genetic algorithm (NSGA-II) used in this article, other optimization methods commonly used in simulations have also been reported in the literature. For example, the design-of-experiments combined with response surface methodology (DOE/RSM) approach [30], the Derringer–Suich desirability function [31] and deep-learning-based surrogate models for optimization [32]. Although the DOE/RSM-based approach has advantages in terms of interpretability, it relies on specific models built through experiments and is suitable for low-dimensional parameter spaces. In contrast, the Derringer–Suich desirability function requires combining multiple objectives into a single objective through weighting, which prevents the independent optimization of each objective. As for deep-learning-based surrogate models for optimization, they typically require extensive finite element simulations to construct the training dataset, resulting in a substantial time cost. Moreover, when the number of objectives increases, the prediction results of deep learning models is not ideal. In contrast, this work evaluates device performance based on a parameterized MEMS+ model, achieving a balance between high simulation accuracy and computational efficiency. Combined with MATLAB-based automated flow and parallel computing, the proposed approach exhibits clear advantages in addressing multi-objective optimization problems for MEMS devices with high-dimensional design parameters.

The proposed method is not only applicable to capacitive accelerometers but can also be extended to other MEMS devices such as gyroscopes and actuators. In the future, all optimized designs will be fabricated and integrated with closed-loop control systems for system-level co-simulation and experimental verification to assess the feasibility of the optimized designs in practical fabrication and circuit.

## Figures and Tables

**Table 1:** Definition, symbol, upper and lower bounds of 22 parameters.

Parameter	Symbol	LB	UB
ProofMassWidth	*W_M_*	1000 μm	3000 μm
ProofMassLength	*L_M_*	3000 μm	3200 μm
VerticalRotorFrameLength	*L* *_V_* *_F_*	600 μm	700 μm
SenseCombFingerWidth	*W_SC_*	5 μm	10 μm
SenseCombFingerLength	*L_SC_*	50 μm	150 μm
SenseCombFingerLargeGapRatio	*R_SC_*	3	20
SenseCombFingerOverlap	*O_SC_*	30 μm	140 μm
SenseCombFingerGap	*G_SC_*	2 μm	6 μm
FeedbackCombFingerWidth	*W_FC_*	5 μm	10 μm
FeedbackCombFingerLength	*L_FC_*	50 μm	150 μm
FeedbackCombFingerLargeGapRatio	*R_FC_*	2	8
FeebackCombFingerOverlap	*O_FC_*	40 μm	140 μm
FeebackCombFingerGap	*G_FC_*	2 μm	6 μm
BeamWidth	*W_B_*	8 μm	15 μm
BeamLength	*L_B_*	600 μm	1050 μm
BeamLengthMiddle	*L_MB_*	45 μm	65 μm
BeamWidthMiddle	*W_LB_*	15 μm	25 μm
CombFingerGroupNumber	*N_C_*	3	6
HorizontalRotorFrameWidth	*W_HF_*	50 μm	90 μm
VerticalRotorFrameWidth	*W_VF_*	50 μm	50 μm
SenseCombAnchorWidth	*W_SA_*	25 μm	25 μm
FeedbackCombAnchorWidth	*W_FA_*	25 μm	25 μm

**Table 2:** Constraints imposed on the design parameters.

Number	Constraint
1	*L_M_* − 4 × *L_V__F_* = 450 μm
2	−*W_M_* – 24 × *L_SC_* + 12 × *O_SC_* – 24 × *L_FC_* + 12 × *O_FC_* <= −2760 μm
3	*W_M_* + 24 × *L_SC_* – 12 × *O_SC_* + 24 × *L_FC_* – 12 × *O_FC_* <= 3760 μm
4	−*L_M_* – 2 × *L_MB_* <= −3154 μm
5	*L_M_* + 2 × *L_MB_* <= 3354 μm
6	*O_SC_*/*O_FC_* >= 0.5
7	*O_SC_*/*O_FC_* <= 1.5
8	*L_SC_* − *O_SC_* >= 10 μm
9	*L_SC_* − *O_SC_* <= 20 μm
10	−*L_FC_* + *O_FC_* <= −6 μm
11	*L_FC_* − *O_FC_* <= 10 μm
12	*L_MB_* − *W_LB_* >= 37 μm
13	*L_MB_* – 2 × *W_LB_* <= 37 μm

**Table 3:** Parameter values of three representative solutions.

Parameter	Symbol	Sensitivity-Oriented Design	Frequency-Oriented Design	Multi-Metric Balanced Design
ProofMassWidth	*W_M_*	2810 μm	1610 μm	1490 μm
ProofMassLength	*L_M_*	3090 μm	3170 μm	3170 μm
VerticalRotorFrameLength	*L* *_V_* *_F_*	660 μm	660 μm	640 μm
SenseCombFingerWidth	*W_SC_*	6 μm	6 μm	6 μm
SenseCombFingerLength	*L_SC_*	93 μm	98 μm	116 μm
SenseCombFingerLargeGapRatio	*R_SC_*	14	12	13
SenseCombFingerOverlap	*O_SC_*	78 μm	83 μm	101 μm
SenseCombFingerGap	*G_SC_*	2 μm	2 μm	2 μm
FeedbackCombFingerWidth	*W_FC_*	6 μm	6 μm	6 μm
FeedbackCombFingerLength	*L_FC_*	150 μm	100 μm	134 μm
FeedbackCombFingerLargeGapRatio	*R_FC_*	4	4	4
FeebackCombFingerOverlap	*O_FC_*	101 μm	92 μm	128 μm
FeebackCombFingerGap	*G_FC_*	2 μm	2 μm	2 μm
BeamWidth	*W_B_*	10 μm	12 μm	10 μm
BeamLength	*L_B_*	897 μm	857 μm	875 μm
BeamLengthMiddle	*L_MB_*	56 μm	49 μm	52 μm
BeamWidthMiddle	*W_LB_*	18 μm	25 μm	20 μm
CombFingerGroupNumber	*N_C_*	3	5	4
HorizontalRotorFrameWidth	*W_HF_*	50 μm	70 μm	90 μm
VerticalRotorFrameWidth	*W_VF_*	50 μm	50 μm	50 μm
SenseCombAnchorWidth	*W_SA_*	25 μm	25 μm	25 μm
FeedbackCombAnchorWidth	*W_FA_*	25 μm	25 μm	25 μm

**Table 4:** Performance comparison between initial design and optimized designs.

Objective	Initial Design	Sensitivity-Oriented Design		Frequency-Oriented Design		Multi-Metric Balanced Design	
*f_y_* (Hz)	1146.06	961.50	−16.1%	1398.80	22.1%	1056.37	−8.1%
C_0_ (pF)	14.59	4.93	−66.2%	9.93	−31.9%	8.64	−40.8%
ΔC (pF)	1.77	1.09	−66.2%	1.02	−42.4%	1.59	−10.2%
F_b_ (g)	7.40	7.88	6.5%	12.31	66.35%	14.23	92.3%
ΔC/C_0_	0.1213	0.2210	82.1%	0.1027	−15.3%	0.1840	51.6%

**Table 5:** Comparison between the simulated and experimental result of the initial design and sensitivity-oriented design.

	Objective	Initial Design	Sensitivity-Oriented Design	
Simulation	Static capacitance	14.59 pF	4.93 pF	−66.2%
	ΔC/C_0_ (reflect sensitivity)	0.1213	0.2210	82.1%
Experiment	Static capacitance	18.78 pF	8.24 pF	−56.1%
	Sensitivity	16.35 mV/g	30.33 mV/g	85.5%

**Table 6:** Results of single-objective optimization.

Number	FOM	|*f_y_* − 1300| (Hz)	C_0_ (pF)	ΔC (pF)	F_b_ (g)
1	0	0	3.47	0.23	6.76
2	0.0037	0.0021	3.26	0.23	7.77
3	0.0049	0.0026	2.65	0.19	7.30
4	0.0059	0.0030	2.66	0.19	6.97
5	0.0106	0.0059	3.27	0.24	7.56
6	0.0109	0.0094	5.60	0.66	7.38
7	0.0144	0.0059	2.08	0.15	5.83
8	0.0146	0.0075	4.07	0.29	7.26
9	0.0155	0.0110	3.75	0.44	6.09
10	0.0163	0.0076	1.98	0.14	6.46

## Data Availability

The original contributions presented in this study are included in the article. Further inquiries can be directed to the corresponding author.
